# Human Peripheral Blood Mononuclear Cell Function and Dendritic Cell Differentiation Are Affected by Bisphenol-A Exposure

**DOI:** 10.1371/journal.pone.0161122

**Published:** 2016-08-10

**Authors:** Alessandra Camarca, Carmen Gianfrani, Fabiana Ariemma, Ilaria Cimmino, Dario Bruzzese, Roberta Scerbo, Stefania Picascia, Vittoria D’Esposito, Francesco Beguinot, Pietro Formisano, Rossella Valentino

**Affiliations:** 1 Institute of Food Science (ISA), National Council of Research (CNR), via Roma 64–83100, Avellino, Italy; 2 Institute of Protein Biochemistry (IBP), CNR, via P. Castellino 11–80131, Naples, Italy; 3 Department of Translational Medical Sciences, Federico II University of Naples, via S. Pansini 5–80131, Naples, Italy; 4 Department of Public Health, Federico II University of Naples, via S. Pansini 5–80131, Naples, Italy; 5 URT “Genomic of Diabetes”, Institute of Experimental Endocrinology and Oncology (IEOS), CNR, via S. Pansini 5–80131, Naples, Italy; Universitatsklinikum Freiburg, GERMANY

## Abstract

Environmental pollutants, including endocrine disruptor chemicals (EDCs), interfere on human health, leading to hormonal, immune and metabolic perturbations. Bisphenol-A (BPA), a main component of polycarbonate plastics, has been receiving increased attention due to its worldwide distribution with a large exposure. In humans, BPA, for its estrogenic activity, may have a role in autoimmunity, inflammatory and allergic diseases. To this aim, we assessed the effect of low BPA doses on functionality of human peripheral blood mononuclear cells (PBMCs), and on in vitro differentiation of dendritic cells from monocytes (mDCs). Fresh peripheral blood samples were obtained from 12 healthy adult volunteers. PBMCs were left unstimulated or were activated with the mitogen phytohemagglutinin (PHA) or the anti-CD3 and anti-CD28 antibodies and incubated in presence or absence of BPA at 0.1 and 1nM concentrations. The immune-modulatory effect of BPA was assessed by evaluating the cell proliferation and the levels of interferon-γ (IFN-γ), interleukin-4 (IL-4), interleukin-10 (IL-10) and interleukin-13 (IL-13) secreted by PBMCs. mDCs were differentiated with IL-4 and GC-CSF with or without BPA and the expression of differentiation/maturation markers (CD11c, CD1a, CD86, HLA-DR) was evaluated by flow cytometry; furthermore, a panel of 27 different cytokines, growth factors and chemokines were assayed in the mDC culture supernatants. PBMCs proliferation significantly increased upon BPA exposure compared to BPA untreated cells. In addition, a significant decrease in IL-10 secretion was observed in PBMCs incubated with BPA, either in unstimulated or mitogen-stimulated cells, and at both 0.1 and 1nM BPA concentrations. Similarly, IL-13 was reduced, mainly in cells activated by antiCD3/CD28. By contrast, no significant changes in IFN-γ and IL-4 production were found in any condition assayed. Finally, BPA at 1nM increased the density of dendritic cells expressing CD1a and concomitantly decreased the expression of HLA-DR and CD86 activation markers. In conclusion, in humans the exposure to BPA causes on PBMCs a significant modulation of proliferative capacity and cytokine production, and on mDCs alteration in differentiation and phenotype. These immune cell alterations suggest that low dose chronic exposure to BPA could be involved in immune deregulation and possibly in the increased susceptibility to develop inflammatory and autoimmune diseases.

## 1. Introduction

A growing body of scientific research has suggested that environmental pollutants, including endocrine disruptor chemicals (EDCs), could interfere on human health, leading to hormonal, immune and metabolic perturbations [1–3]. EDCs, particularly with estrogenic activity, can work in a tissue specific manner, particularly when the exposure occurs during development [4]. EDCs structure, in fact, is similar to endogenous steroid hormones, including estrogen (E2) and androgen, and so they can bind the corresponding hormone receptors, with agonist or antagonist activity. The immune system is sensitive to EDCs particularly during prenatal period, due to their potent modulatory activity on cellular immune responses [3,5,6]. Among EDCs, bisphenol-A (BPA) has been receiving increased attention, for its xeno-estrogenic activity and its large environmental distribution, mainly in modern fast-food/processed/packaged food diet, beverages, thermal paper, air, water and soil. In fact, as main plastic component, BPA is one of the highest volume chemicals produced globally and it is able to accumulate in adipose tissue during the whole life [7–10]. Although BPA has a short life, due to the instability of BPA-based polymers, humans are chronically exposed to low, but still active, doses already during fetal or neonatal development, a critical window for the “fetal basis of adult disease” [11–13]. Considering that BPA is able to cross placenta and to concentrate in amniotic fluid, its effect on organ development and differentiation is dependent not only on the dose, but also on the early prenatal exposure [7,12,13]. In addition, BPA effect remains chronic during adult life and that is confirmed by detectable amount assayed in plasma and urine of several populations [10,13–16]. At environmental low doses, such as 1nM (0.23ng/ml), dose in the range of human serum concentrations [10,12,16], BPA, alone or in combination with other chemical compounds, may stimulate several cellular responses both in humans and in animals, with reproductive and metabolic defects [11,13,17,18]. For instance, BPA is able to impair obesity-related pathways by altering cell signaling involved in weight and lipid homeostasis and by enhancing and altering adipogenesis and lipogenesis with generation of dysfunctional adipocytes, both in animals and in humans [19–23]. For its hormone-like effects, besides interference on neurological development, reproduction and hormonal regulation in wildlife and humans, BPA may give rise to systemic low-grade inflammation, with adverse metabolic health consequences late in adulthood, such as insulin resistance, metabolic syndrome, diabetes and cardiovascular diseases [11–13,16,22–26]. These effects may act through the regulation of hormonal and metabolic signaling, mainly mediated by nuclear receptors or other still unknown mechanisms, with interferences on several systems [27].

Besides the trigger of obesity and related metabolic and immune-diseases [28–30], it has been proposed for BPA a role in the loss of self-tolerance, autoimmunity, inflammatory and allergic diseases, a growing phenomenon in the industrialized countries, via the modulation of immune responses [31–33]. This hypothesis is supported by recent epidemiological studies showing that higher urinary BPA concentrations were associated with increased asthma risk in children [34], and with allergy-related asthma in females [35]. A link between BPA exposure and multiple sclerosis (MS) was also suggested [30,31].

In animal models, the detrimental effect of BPA was suggested by several studies, demonstrating that BPA may affect T cell proliferation [36–38] and Th1/Th2 polarization, although with contrasting results [39–42]. Notably, it has been found that in mice the prolonged exposure to BPA stimulates the proliferation and differentiation of splenocytes in a Th1 phenotype with increase in IFN-γ and decrease in IL-4 production compared to control mice, but also modulates the thymocyte functions [36–40]. At variance, a different study has reported that higher doses of BPA induce a Th2 cell polarization with increase of IL-4 and IL-10 [41], although very little is known regarding the BPA effect on the differentiation and function of dendritic cells, the most powerful antigen-presenting cells of the immune system [43].

Nevertheless, these studies have used high amount of BPA (μM), compared to environmental doses and little is known on the effect of BPA on human immune components, including effector cells but also antigen presenting cells.

To date, the mechanisms by which BPA exerts all these biological actions are still not fully understood and, unfortunately, the large body of studies have been done in animal models, being very few data available in human immune systems [27].

In this context, also considering the controversial reports on the implications of BPA in human health, aim of this study was to check the effects of a short time exposure to very low concentration of BPA on proliferation, differentiation and function of immune cells. To address these aspects, we used freshly isolated peripheral blood mononuclear cells (PBMCs) and monocytes-derived dendritic cells (mDCs) from adult healthy volunteers, at basal condition and after mitogen activation.

## 2. Materials and Methods

### 2.1. Chemical reagents and antibodies

Bisphenol-A (BPA) supplied by Sigma-Aldrich Chemical Co. (St. Louis, MO, USA). in ethanol was a generous gift of Prof. C. Crescenzi (Department of Pharmaceutic Science, University of Salerno, Italy). Aliquots of BPA were prepared in ethanol at 100 nM concentration and kept at -20°C until the use.

Phytohaemagglutinin (PHA) was purchased from Roche (Milan Italy). Lipopolysaccharide (LPS) was from Sigma-Aldrich Chemical Co. (St. Louis, MO, USA). Purified anti-CD3 MAb (Orthoclone OKT3) was from Janssen-Cilag, (Milan, Italy) and anti-CD28 from BD Pharmingen (San Diego, CA, USA).

RPMI 1640, human AB sera, non-essential AA, sodium pyruvate, L-glutamine and antibiotics were all provided by BioWhittaker-Lonza (Verviers, Belgium). Human recombinant IL-4 and GM-CSF were from Miltenyi Biotech (Bologna, Italy).

Both capture (purified) and detection (biotinylated) anti-IFN-γ, anti-IL-4, anti-IL-10 and anti-IL-13 monoclonal Abs were from MabTech (Nacka Strand, Sweden). Conjugated antibodies for FACS analysis were purchased as follow: PerCP-anti-CD3, PerCP-anti-HLA-DR and PE-anti-CD11c from Miltenyi Biotech, FITC-anti-CD14 from eBioscience (San Diego, CA), PE-anti-CD86 and FITC-anti-CD1a from BD Pharmingen (San Diego, CA, USA).

### 2.2. Blood sample collection, isolation of peripheral blood mononuclear cells (PBMCs) and functional assays

Fresh peripheral blood samples were obtained from 12 (7 males and 5 females) healthy, normal weight individuals with an average age of 36.1 ± 14.5 years (range 26–61 years). All donors gave written informed consent to the study. The procedure was approved by the Ethical Committees of Hospital Moscati, Avellino (OsSc registry n. 06/09, 12/21/2007). Twenty ml of uncoagulated blood samples were collected and PBMCs were isolated by Ficoll gradient according to the manufacturer’s instructions within 2 hours after blood withdrawal. After washing, cells were suspended in RPMI 1640 containing 10% heat-inactivated, human AB sera, non-essential AA, sodium pyruvate, L-glutamine and antibiotics at a density of 2.5x10^5^ in 250μl/well for functional assays. Cells were left unstimulated or stimulated with PHA (0.5μg/mL) or with immobilized anti-CD3 (10μg/mL) and soluble anti-CD28 (1μg/ml) antibodies. We performed experiments in absence or in presence of BPA at different concentrations (0.1 and 1nM) in U bottom 96-well plates, in triplicates. After 48 hours, 200μl of cell supernatants were harvested for cytokine determination. The remaining cells were pulsed with 0.5 μCi [3H]-thymidine (Amersham Pharmacia, Milan, Italy) and incubated for additional 16 hours before the [3-H]-thymidine incorporated in the DNA was measured by liquid scintillation counting (Top-Count, Packard, Canberra).

The levels of interferon-γ (IFN-γ), interleukin-4 (IL-4), interleukin-10 (IL-10) and interleukin-13 (IL-13) were measured by ELISA method in supernatants, by standard sandwich assay, as previously described [44]. Sensitivity of ELISA assays were as follows: 31pg/mL for IFN-γ, 9pg/mL for IL-4 and IL-10, and 62pg/mL for IL-13.

### 2.3. Differentiation of monocyte–derived dendritic cells (mDCs), flow cytometer analysis and mixed lymphocyte reaction (MLR)

To generate mDCs a standard procedure was applied [45]. Buffy coats of blood were obtained from 6 healthy normal weight donors, all males, afferent to the Blood Transfusion Center (Azienda Ospedaliera S.G. Moscati, Avellino, Italy), after a written informed consent. PBMCs were isolated by density gradient centrifugation. Cells were washed, suspended in complete medium (RPMI1640+5%HS, plus supplements) and incubated at 10x10^6^ concentration in 3 ml in 6 well plates for 2 hours. Non-adherent cells were then removed by gently washing, and fresh medium containing rhGM-CSF and rhIL-4 (both at 100 U/ml) was added, in absence or in presence of 1nM BPA. Cells were incubated for 6 days, thereafter cells were harvested, analyzed by flow cytometry (FACS) and the differentiation culture medium was stored at -80°C for multiplexing cytokines/chemokines analysis. Next, mDCs (2x 10^5^ cells) were analyzed for the expression of CD11c, CD1a, CD86 and HLA-DR using a BD FACSCalibur and Cell Quest software. In all cases the purity of mDCs was >90%.

For mixed lymphocyte reaction (MLR) experiments, mDCs from three different samples were differentiated as described above and subsequently matured by adding LPS. At day 6, 1ml of culture medium was replaced with fresh medium containing LPS (10 μg/ml) and BPA (1nM) and cells were incubated for additional 24 hours. Mature DCs were then collected, analyzed by flow cytometer and co-incubated with allogenic non-adherent fraction of PBMCs (1x10^4^ DCs: 5x10^4^ NA-cells), in 200 μl of complete medium in 96 well plates. A total of 5 MLRs was set up, in triplicates. IFN-γ production was evaluated after 48 hours by ELISA assay.

A panel of 27 different cytokines, chemokines and growth factors produced by immature mDCs was determined in the culture supernatants using BIOPLEX multiplex Human Cytokine and Growth Factor assay kit (Bio-Rad, Hercules, CA,USA), according to the manufacturer’s instruction.

## 3. Statistical Analysis

All data were expressed as median [min max] due to the skewed distribution of the studied variables. Accordingly, the non-parametric Friedman test, followed by the Wilcoxon test for dependent samples, was used to asses the significance of the effect of BPA at different concentrations compared to untreated cells. For MLR experiments only, differences were evaluated by paired T-test. All tests were two sided and a p value <0.05 was considered statistically significant with no adjustment for multiple comparisons. All analyses were performed using the statistical computing environment R (R Foundation for Statistical Computing, Vienna, Austria).

## 4. Results

### 4.1. BPA stimulates the proliferation of peripheral blood mononuclear cells (PBMCs)

**Fig 1:**
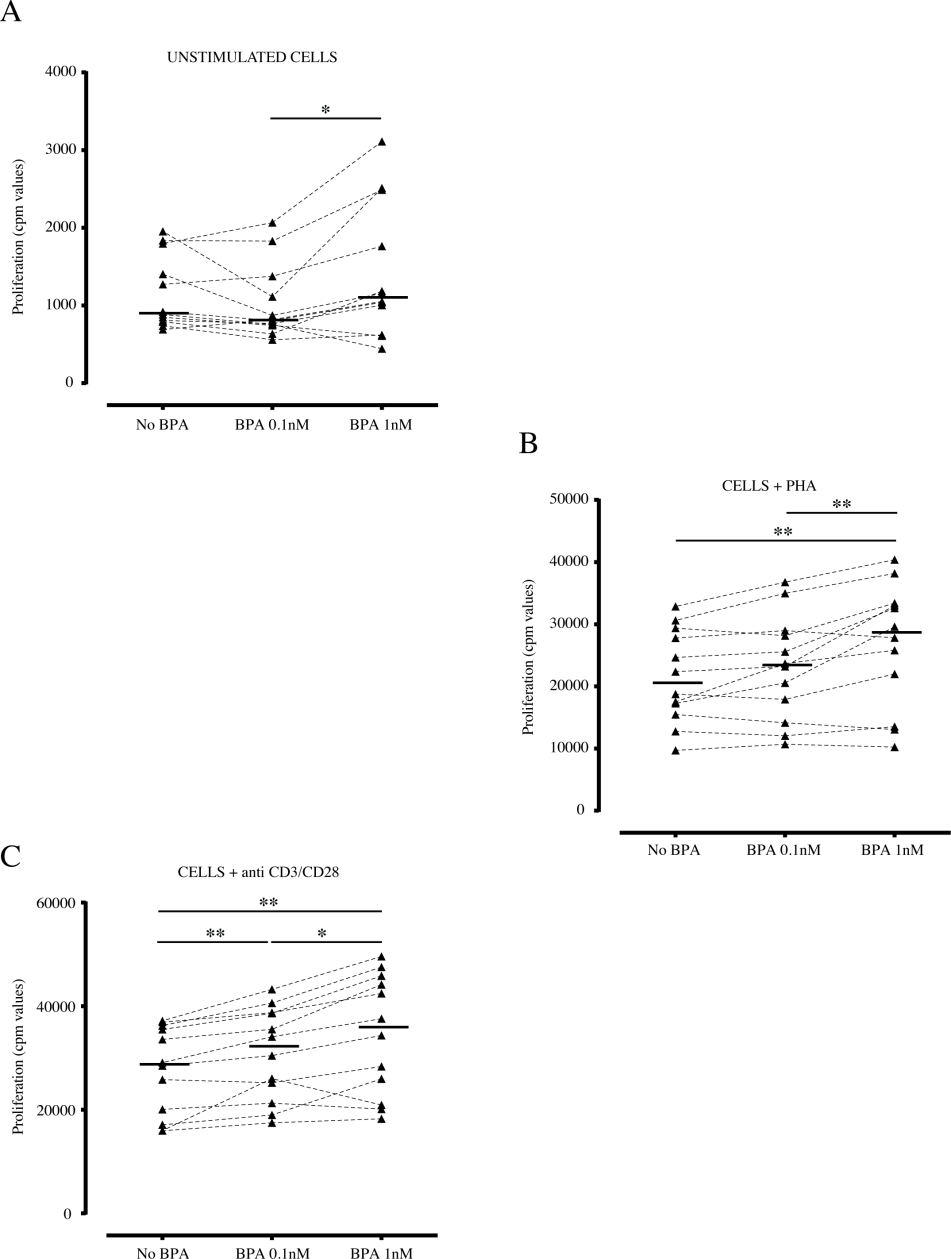
Effect of BPA on PBMCs proliferation. Freshly isolated PBMCs (n = 12) were incubated in absence or in presence of BPA at the indicated concentrations (0.1 and 1nM). After 48 hours of incubation, cells were pulsed with [3-H]-thymidine for 16 hours before the harvesting. Results were expressed in cpm (count per minute). Experiments were performed on (A) unstimulated PBMCs or (B) under stimulation with phytohemagglutinin (PHA; 0.5μg/mL), or (C) with immobilized anti-CD3 (10μg/mL) and soluble anti-CD28 (1μg/ml) antibodies. Each dot represents mean value of triplicate wells. Bars indicate median values (n = 12). Asterisks indicate statistically significant differences (*p<0.05 and **p<0.01) by non-parametric Friedman test, followed by the Wilcoxon test for dependent samples.

In order to address the possible effects of BPA (0.1nM and 1nM) on PBMCs proliferation, we performed experiments on cells from 12 adult healthy donors, by brief exposure either at steady state condition or under polyclonal mitogens, as PHA and anti-CD3/CD28 stimuli (Fig 1). In all three analyzed experimental conditions (unstimulated, anti-CD3/CD28, and PHA stimulated cells), we observed a dose-dependent increase in cell proliferation in presence of BPA, compared to untreated cells. In fact, basal cell proliferation significantly increased at the higher 1nM BPA concentration (Fig 1A). The same trend was found when cells were stimulated with PHA or anti-CD3/CD28 (Fig 1B and 1C). In particular, in cells stimulated with PHA, cell proliferation was enhanced significantly at 1nM of BPA concentrations (p<0.01) (Fig 1B). Similarly, in cells activated via T-cell receptor (TCR), through the anti-CD3/CD28 pathway activation, the proliferation significantly increased at both 0.1 and 1nM BPA (p<0.01) (Fig 1C). Interestingly, in all three stimulatory conditions, the proliferative rate observed at 1nM BPA was significantly higher than that obtained at the lower 0.1nM dose (p<0.05 and p<0.01).

### 4.2. BPA inhibits the production of IL-10 and IL-13 by peripheral blood mononuclear cells (PBMCs)

With the aim to address if BPA affects the secretion and the balance of Th1/Th2 cytokines, we analyzed the production of IFN-γ, IL-10, IL-13 and IL-4, by PBMCs incubated as described above.

The analysis of the IFN-γ did not show significant changes when BPA was added to the cell cultures in all experimental conditions, although a trend to an increased production was observed in some subjects (data not shown).

**Fig 2:**
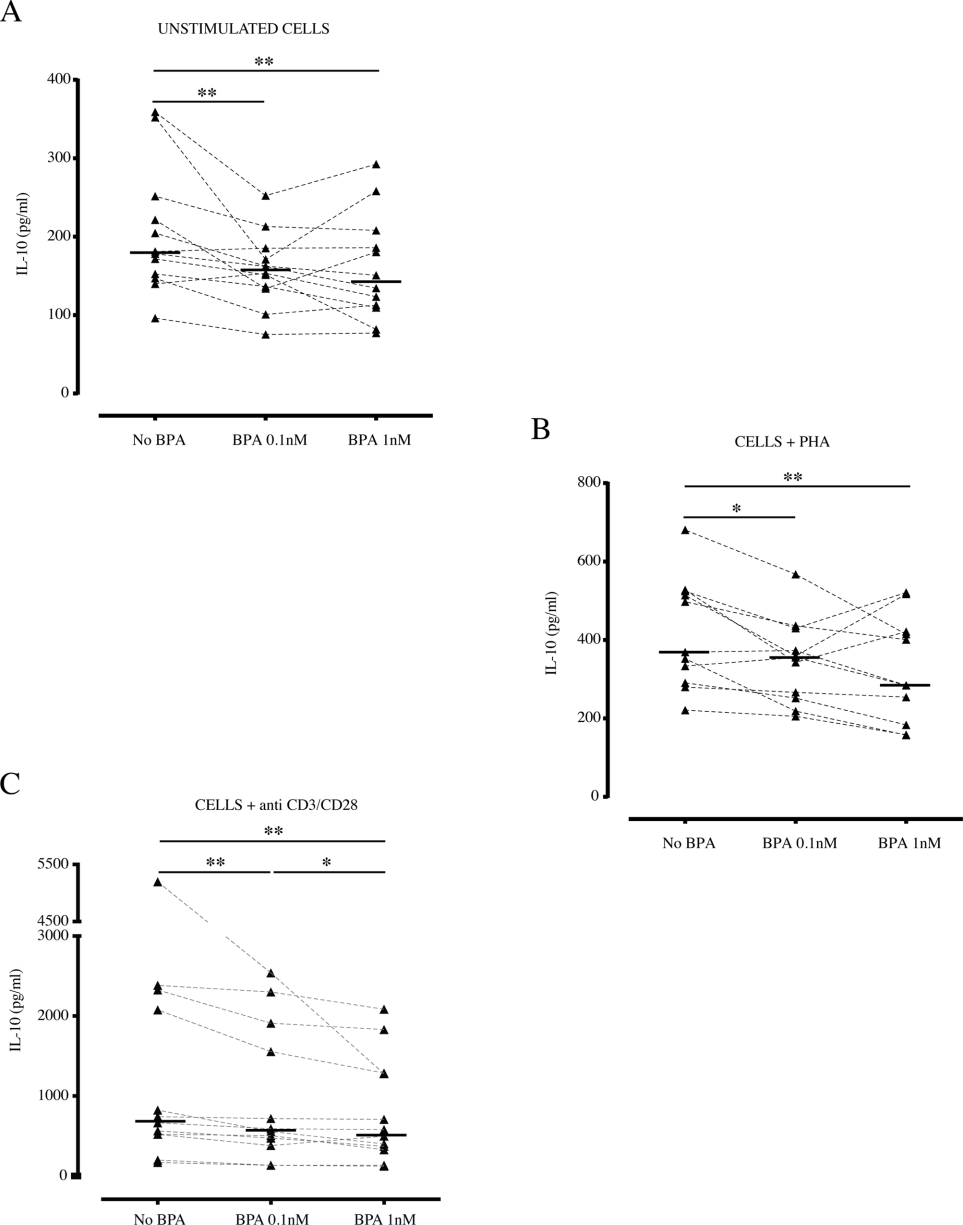
Effect of BPA on IL-10 production by PBMCs. Freshly isolated PBMCs (n = 12) were incubated in absence or in presence of BPA at the indicated concentrations for 48 hours. Cell culture supernatants were collected, assayed for IL-10 by ELISA and expressed in pg/ml. (A) Effect of BPA on IL-10 production by unstimulated cells. (B) Effect of BPA on IL-10 production by PBMCs activated with PHA. (C) Effect of BPA on IL-10 production by PBMCs activated with anti-CD3/CD28 antibodies. Each dot represents mean value of triplicate wells. Bars indicate median values (n = 12). Asterisks indicate statistically significant differences (*p<0.05 and **p<0.01) by non-parametric Friedman test, followed by the Wilcoxon test for dependent samples.

**Fig 3:**
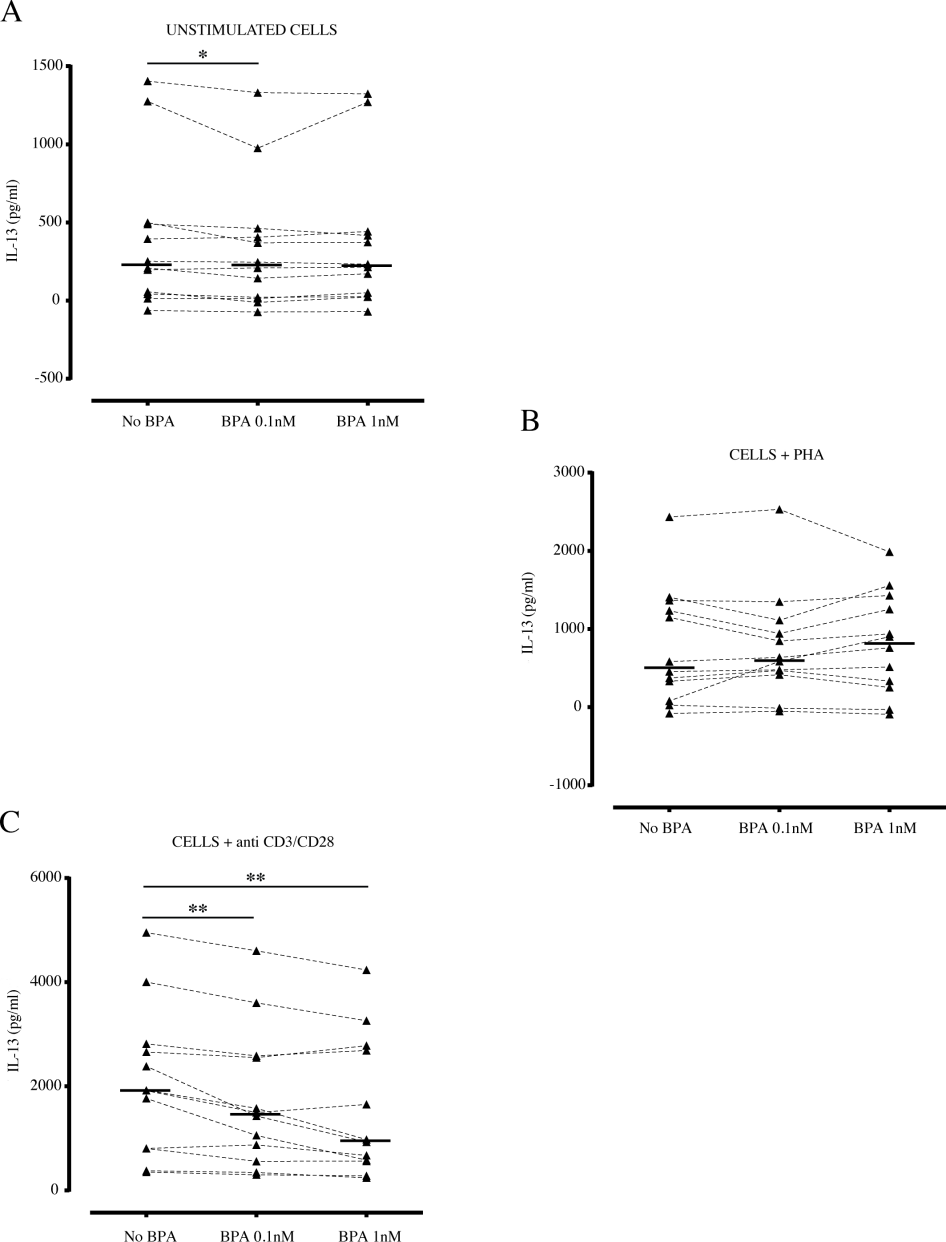
Effect of BPA on IL-13 production by PBMCs. Freshly isolated PBMCs (n = 12) were incubated as described in absence or in presence of BPA and cell culture supernatants were assayed for IL-13 production by ELISA, after 48 hours, and expressed in pg/ml. (A) Effect of BPA on production of IL-13 by unstimulated cells. (B) Effect of BPA on IL-13 production by PBMCs activated with PHA. (C) Effect of BPA on IL-13 production by PBMCs activated with anti-CD3/CD28 antibodies. Each dot represents mean value of triplicate wells. Bars indicate median values (n = 12). Asterisks indicate statistically significant differences (*p<0.05 and **p<0.01) by non-parametric Friedman test, followed by the Wilcoxon test for dependent samples.

Regarding the BPA effect on IL-10 production by PBMCs, a significant decrease was evident both in unstimulated and in PHA and anti-CD3/CD28 stimulated cells compared to untreated cells, already at 0.1nM BPA concentration (p<0.01) (Fig 2A, 2B and 2C). The secretion of IL-10 was further reduced in presence of 1nM BPA upon stimulation with anti-CD3/CD28 (Fig 2C), suggesting, also for IL-10, a dose-dependent effect for this chemical compound. Concerning the production of Th2 cytokines, a low but significant decrease in IL-13 secretion was shown in unstimulated condition, when cells were incubated with BPA 0.1nM (p<0.05) (Fig 3A). Similar results were obtained with BPA 1nM, although the statistical significance was not reached. At variance, BPA did not affect significantly the IL-13 production obtained under the effect of PHA (Fig 3B**)**. Conversely, a stronger effect of BPA was found in cells activated by anti-CD3/CD28, showing a sensitive BPA reduction of IL-13 production (p<0.01) (Fig 3C).

Results on the IL-4 production were less clear, since this cytokine was detected at very low levels in different samples. Collectively, IL-4 seems not to be affected by the BPA incubation in any of the cell culture conditions assayed (data not shown).

### 4.3. BPA affects monocyte–derived dendritic cells (mDCs) differentiation and function

**Fig 4:**
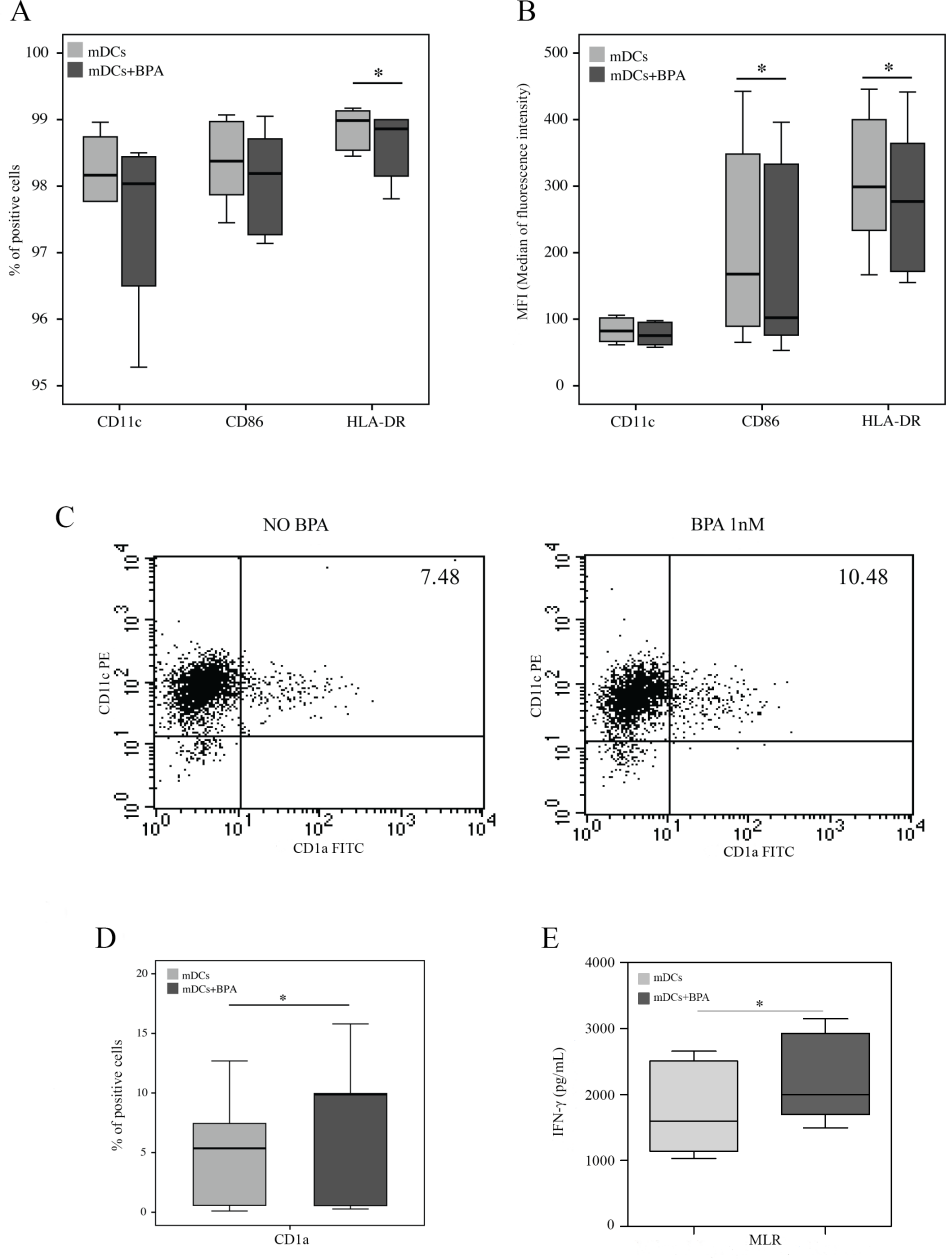
BPA affects the phenotype of monocyte derived Dendritic Cells (mDCs). Dendritic Cells (mDCs) (n = 6) were differentiated from PBMCs for 6 days in absence or in presence of BPA at 1nM concentration and then analyzed by flow cytometer. (A) Percentages on total mDCs of cells expressing CD11c, CD86 and HLA-DR, (B) Median of fluorescence intensity of CD11c, CD86 and HLA-DR on mDCs. (C) Examples of staining for CD11c and CD1a on mDCs differentiated with or without BPA. (D) Percentages on total mDCs of cells expressing CD1a. (E) Mixed Lymphocyte Reactions (MLR) (n = 5) were performed with mDCs differentiated and subsequently LPS-matured in presence or absence of BPA, and co-incubated with allogenic non-adherent fractions of PBMCs (1:5 ratio mDCs:NA-cells). Experiments were performed in triplicates and IFN-γ secretion was evaluated after 48 hours. In panels A, B, D and E we reported median and ranges [min max] of the analyzed samples. Asterisks indicate statistically significant differences (*p<0.05) by non-parametric Friedman test, followed by the Wilcoxon test for dependent samples, except for MLR experiments, where paired T-test was applied. In panels A, B, D and E we reported median and ranges [min max] of the analyzed samples. Asterisks indicate statistically significant differences (*p<0.05) by non-parametric Friedman test, followed by the Wilcoxon test for dependent samples.

Dendritic cells were differentiated from peripheral blood adherent monocytes obtained from 6 healthy donors. We checked the capability of mDCs maturation by evaluating the increased expression of CD80, CD86 and HLA-DR after LPS treatment (data not shown). Immature mDCs were incubated in presence or absence of BPA 1nM for 6 days and then analyzed for the expression of differentiation and maturation markers. No difference was found in the cell recovery between untreated and BPA-treated mDCs, as the number of cells (reported as mean ± SD) was: 6.04 ± 4.2x10^5^ in no-BPA cultures and 6.72 ± 2.9x10^5^ in BPA-treated cultures, thus excluding a toxic effect on cell viability during the in vitro differentiation phase. Moreover, no significant differences were in the percentage of differentiated mDCs, as the densities of CD11c^+^CD14^-^ cells (evaluated as mean ± SD) was: 97 ± 0.15% in no-BPA cultures and 96.2 ± 1.86% in BPA-treated cultures. Conversely, the exposure to BPA significantly decreased the percentage of cells expressing the HLA-DR (p<0.05) (Fig 4A). When we monitored the median of fluorescence intensity of CD11c, CD86 and HLA-DR molecules on cell surface, immature mDCs, differentiated in presence of BPA, showed a significant reduced expression of both CD86 [MFI median and ranges: no BPA 167.8 (65.2–442.5), BPA-treated 102.2 (53.1–396), p<0.05] and HLA-DR [MFI median and ranges: no BPA 299 (167–445), BPA-treated 277 (155–441), p<0.05] (Fig 4B). In contrast, the expression of CD1a (Fig 4C and 4D) was significantly increased (p<0.05) upon the effect of BPA. In particular, although we found a large variability in the percentage of CD1a positive cells among the analyzed donors [40] [percentage of median of CD1a positive cells: no-BPA 5.36 (range 0.1–12.7); BPA-treated 9.88 (range 0.3–15.8)], a consistent increment was observed in all six analyzed samples upon BPA treatment (p<0.05) (Fig 4C and 4D). Interestingly, almost all CD1a positive mDCs co-expressed CD11c, CD86 and HLA-DR, and the immune-modulatory influence by BPA was detected in all CD1a positive cell subsets (data not shown).

In order to investigate if the mDCs phenotypic changes by BPA may have a biological consequence on T cells, we performed MLR experiments on mDCs from three healthy donors, differentiated and subsequently LPS-matured in presence or absence of BPA, and then co-incubated with allogenic non-adherent fractions of PBMCs. Results showed that BPA-treated mDCs were able to significantly increase the IFN-γ production (p<0.05), thus indicating that BPA can enhance the pro-inflammatory reaction in MLRs (Fig 4E).

Finally, we analyzed the immature mDCs culture media, looking at possible differences in several soluble factors, accumulating during the differentiation period (Table 1). Results showed no significant differences among untreated and BPA-treated mDCs, although a trend in increase of some pro-inflammatory cytokines, such as Interleukin-6 (IL-6) and Monocyte Chemoattractant Protein-1 (MCP-1), and a decrease in some anti-inflammatory factors, for example Interleukin-1 receptor antagonist (IL-1ra), was observed.

**Table 1:** Cytokines and Growth Factors released by dendritic cells, expressed as pg/ml.

Bio-Plex Panel	CTR	BPA 1nM
IL-1b	3.3 ± 2	3.3 ± 2
IL-1ra	508.2 ± 200	384.5 ± 66.3
IL-2	18.1 ± 3.3	16.8 ± 1
IL-4	3311.2 ± 1482	5829.8 ± 172.5
IL-5	0.8 ± 0.005	0.8 ± 0.04
IL-6	17.9 ± 7.6	24 ± 10.7
IL-7	2 ± 0.2	2.04 ± 0.3
IL-8	385.6 ± 89.3	337.8 ± 90.2
IL-9	19 ± 7.6	17.7 ± 4.3
IL-10	11.6 ± 2.7	11 ±1.4
IL-12	14 ± 1.4	14.05 ± 0.8
IL-13	1.9 ± 0.3	2 ± 0.3
IL-15	20.9 ± 3.2	20.3 ± 2
IL-17	36.2 ± 11.4	35.7 ± 7.3
EOTAXIN	27.4 ± 5.5	26.1 ± 1.2
FGF basic	27.4 ± 5.9	24.6 ± 1.1
G-CSF	42.7 ± 6.6	43.7 ± 8.5
GM-CSF	ND	ND
IFNγ	147.7 ± 34.9	138.5 ± 9.7
IP-10	701.5 ± 415	705.2 ± 432.9
MCP-1	642.1 ± 137.2	1037.3 ± 340.8
MIP-1a	7.3 ± 2.1	8.6 ± 6.3
MIP-1b	302.8 ± 208.8	259.8 ± 152.8
PDGF-bb	31.5 ± 4.4	33.3 ± 16.7
RANTES	70.9 ± 49.3	73.1 ± 44.5
TNF-a	35.7 ± 9.6	33.4 ± 1.9
VEGF	43.5 ± 8.4	41.5 ± 3.5

## 5. Discussion

An increasing number of studies has associated the environmental BPA exposure to the recent changes in prevalence of the allergic diseases, including food allergies and/or loss of self-tolerance leading to autoimmunity and gastrointestinal infections [33,46–53].

BPA, as endocrine disruptor with natural estrogen activity, at small doses can cause different effects from those generated at higher doses, with an oscillating non-monotonic dose response. Numerous evidences, however, have shown that BPA can have significant metabolic and immune effects at environmental low doses, which may not be apparent at higher doses used in traditional toxicological studies [13,54,55]. For example, at micromolar doses, the toxic effect on immune cells could regulate expression of a distinct set of genes involved in growth and development, different from those regulated at low doses [13,54,55]. In particular, Gostner et al. [55] reported an inhibitory effect of BPA on T-cell proliferation, with immunosuppressive effects. The BPA concentrations applied in this study (0.1 and 1nM) are widely considered as “low-dose” used for in vitro experiments and are consistent with human chronic exposure [11,13,56,57], with different effects on cell proliferation.

Moreover, BPA ingested with food and beverage is absorbed in gastrointestinal tract and partially metabolized in the liver by uridine 5’-diphospho-glucuronosyl-transferase (UDP-UGT) enzyme [18,22,57] Subsequently, BPA has the first contact in the gut where the great majority of immune competent cell is localized, with possible consequences on the immune response.

In this in vitro study, we showed that BPA, at concentrations comparable to those in human serum [7,9,10,13], might affect the human immune system homeostasis and reactiveness to external stimuli, by altering both peripheral blood mononuclear cells (PBMCs) and monocyte-derived dendritic cells (mDCs) function. In particular, BPA increased significantly PBMCs proliferation, mainly after cell-activation with PHA and anti-CD3/CD28. These results are in agreement with those observed in murine immune system cells, where it has been described that the exposure to high doses (20μM) of BPA increases the proliferation of concanavalin A-stimulated splenocytes [36] and of thymocytes [37], while analogous results were found also in goldfish [38]. By contrast Gostner et al. [55] found a decreased cell viability in human PBMCs treated with micromolar doses of BPA, as well as other studies [54] reporting an immune-suppression activity of high doses of the chemical in lupus-prone murine models, thus confirming a broad, non-monotonic, BPA effects on immune cells.

In mouse models, it has also been shown that BPA affects the Th1/Th2 balance, though with conflicting results. In fact, some studies found that BPA exposure induced differentiation of splenocytes in a Th1 phenotype with increase in IFN-γ and decrease in IL-4 production, compared to control mice [39,40,58]. At variance, a different study reported that BPA induce a Th2 cell polarization with increase of IL-4, IL-10 and IL-13 in adult mice [41,42,59], whilst the prenatal exposure to BPA up-regulated both Th1 and Th2 immune response in the same animal model [59]. Interestingly, the percentages of T regulatory function were decreased in both groups exposed to BPA [59].

Regarding cytokine production by PBMC, we found that BPA, at environmental doses, significantly decreased IL-10 secretion in all analyzed experimental conditions, suggesting a subsequent reduction in anti-inflammatory immune responses. In fact, IL-10, classified as a class-2 cytokine, has a relevant effect on the homeostasis of immune response, with a crucial role in limiting the immune response to pathogens, in the establishment of the oral tolerance, and in preventing inflammatory and autoimmune pathologies [50–53]. Being secreted by many cell types, including Th2 and regulatory T cells, B cells, NK, mast cells, macrophages, monocytes and dendritic cells, IL-10 blocks immune responses at different levels by acting directly and indirectly on both the innate and adaptive arms of the immune system. Consequently, IL-10 is able to inhibit the production of pro-inflammatory Th1 cytokines by effector T cells, cell proliferation, and the expression of MHC-class II, and co-stimulatory molecules on antigen presenting cells [46,48–50,60,61]. Thus, the reduced capacity of PBMCs to secrete IL-10 under BPA exposure, can have important implication on tolerant/suppressive immune responses, and might act as inducer factor of pro-inflammatory pathways involved in autoimmune and inflammatory diseases. However, to better clarify the IL-10 involvement in increasing cell proliferation upon BPA treatment, further experiments are needed.

Similar results were obtained on IL-13 secretion by PBMCs, resulting decreased upon BPA treatment. In particular, when cells were activated by anti-CD3/CD28 antibodies, we found a BPA dose-dependent reduction of IL-13. Conversely, spontaneous IL-13 remained almost unchanged, whilst IL-13 after stimulation with PHA tended to increase. These results are not surprising since, in our experimental system, IL-13 was secreted almost exclusively by Th2 cells, and it has been reported that activating T cells with different stimulus may result in different cytokine production [62]. Concerning the possible consequences of IL-13 reduction by BPA, it is well known that IL-13, together with IL-4, orchestrate type-2 immunity, inducing host protection, with defense mechanisms against parasites and controlling infection. In fact, IL-13 is critical to combat the gut infestation, such as various intestinal nematode infections, due to its capability to produce mucus, to increase the proportion of goblet cell and worm expulsion, but also by increasing immunoglobulin E (IgE) production and gut motility [53,63–65]. In addition, IL-13, activating B cells, is a key mediator of allergic disease and asthma [34,35,64] and, in fact, it is strongly increased in these abnormal immune responses.

The BPA-dependent IL-13 reduction observed in our study seems not to confirm a Th2 shift of immune response previously shown in mice models exposed to BPA. Accordingly, we found that IL-4 remained unchanged upon BPA exposure. Nevertheless, our finding that BPA reduced IL-13 secretion could be responsible for an altered inflammatory reaction to helminthic infestation in the gut, with subsequent deleterious effects on gut immune function. In fact, a similar effect was reported in young rats prenatally exposed to BPA, showing an inappropriate Th1/Th2 cytokines profile in infected intestinal mucosa, with impaired cellular response to food antigens, and increased susceptibility to intestinal parasitic infection in the juveniles [53].

Finally, concerning the development and function of dendritic cells, the chief orchestrator of immune responses, BPA exposure during the in vitro differentiation from peripheral blood monocytes resulted in a marked change of mDC phenotype. Immature mDCs generated in the presence of environmental low dose (1nM) of BPA and analyzed at the end of the differentiation period, expressed a reduced level of CD86 and HLA-DR, thus suggesting a down-regulation of the antigen-presenting capacity of dendritic cells [66]. In this regard, only two studies have addressed the effect of BPA on *in vitro* mDCs maturation, with conflicting results. In particular, Guo et al. [66] showed on human mDCs, that BPA (100nM), together with TNF-alpha, increased the expression of CD1a and HLA-DR, but not of CD80, CD86 and CD83. Conversely, Pisapia et al. [43] have also described that high doses of this chemical (10μM) up-regulates CD11c, HLA-class II and CD86 on mouse bone marrow derived DCs. Thus, the difference in the BPA concentration and the fact that we analyzed the mDC phenotype in absence of maturation stimuli, can explain the discrepancies between our results and those obtained in previous studies.

Interestingly, BPA-treated mDCs evidenced a significant increase in CD1a expression, a marker of differentiation, whose biological significance is still debated, also considering the extremely variability in its expression on humans mDCs [67–69]. Of note, it has been reported that CD1a expressing DCs derived from peripheral blood monocytes are able to produce IL-12p70 and to polarize naive CD4 T cells to a Th1 phenotype [68], thus suggesting a pro-inflammatory effect of BPA. In line with this hypothesis, although preliminary, our data indicated that BPA-treated mDCs were able to enhance the IFN-γ production in MLRs.

Interestingly, although no significant variation in cytokine, chemokine and growth factor secretion was found, IL-6 and MCP-1 secretion, well-known pro-inflammatory cytokines involved in the etiology of obesity- and diabetes-related diseases [26], seemed to be slightly increased upon BPA treatment, while IL-1ra, with anti-inflammatory activity, a natural inhibitor of the pro-inflammatory effect of IL1β, had a trend in reduction. We are aware that, to better define this potential pro-inflammatory role of BPA on mDCs, these data must be confirmed in further experiments with a larger number of samples. Anyway, considering the relevant role of mDCs in either the initiation and progression of innate and adaptive immunity, with strategic location throughout peripheral tissues [70], the alteration of mDCs function by chronic low-dose BPA exposure could have a potential deleterious impact on immune responses. Taken together, the increase in cell proliferation and the decrease in IL-10 production, as well as the up-regulation of CD1a on dendritic cells, could indicate a pro-inflammatory effect of BPA on human immune responses. Nevertheless, since we did not find a substantial effect on IFN-γ production, further investigation are needed to confirm a possible Th1 immune-response polarization. In addition, the decreased IL-13 can be particularly important in the gut immune defenses.

We are confident that the limitation of this study is the lack of evidences on the mechanisms by which BPA exerts its biological action, still unclear, enigmatic and not fully characterized.

In the literature, BPA, as “imperfect” estrogen, is reported to bind Estrogen Receptor (ER) isoforms, with modulation of immunity [27, 71–76]. In particular, BPA may act with different effects compared to estrogen, binding the classic estrogen receptors ERα and ERß (genomic pathways), but mainly acting *via* non genomic responses, through non-classic estrogen receptors, such as estrogen-related receptor (ERRs) [77] and the trans-membrane G protein-coupled estrogen receptor 1 (GPR30/GPER) [72]. These are reported to be the major sex-steroid receptors present in human monocytes, and their expression is not modified during differentiation in vitro and in vivo [76], but with influence on regulation and expression of key-genes involved in development and homeostatic hormone signalling pathways. The relationship between GPR30 activation and increased concentration of Epidermal Growth Factor (EGF) and EGFR auto-phosphorylation supports this hypothesis and is related to cell growth, proliferation and differentiation. Moreover, intriguing is the possibility that BPA may activate inflammatory pathways in several tissues via innate Pattern Recognition Receptors (PRRs), such as Toll-like Receptors (TLRs), pathogen receptors involved in mediating the inflammatory response to metabolic stress and a critical link between intestinal microbiota and host metabolism [78].

In conclusion, we have confirmed that in adult individuals BPA chronic exposure may affect the function of immune competent cells PBMCs and mDCs, causing a deregulation of either T effectors and of regulatory cell subset. Thus, in combination with other insults over a lifetime, may increase the risk to develop immune diseases in adulthood.
